# The effect of cognitive fatigue on prefrontal cortex correlates of neuromuscular fatigue in older women

**DOI:** 10.1186/s12984-015-0108-3

**Published:** 2015-12-21

**Authors:** Ashley E. Shortz, Adam Pickens, Qi Zheng, Ranjana K. Mehta

**Affiliations:** Department of Environmental and Occupational Health, School of Public Health, Texas A&M University, College Station, TX 77843 USA; Department of Epidemiology and Biostatistics, School of Public Health, Texas A&M University, College Station, TX 77843 USA

**Keywords:** Physical fatigue, Near infrared spectroscopy, Cerebral oxygenation, Neuroergonomics, Stroop Color Word, n-back, Cognitive demand

## Abstract

**Background:**

As the population of adults aged 65 and above is rapidly growing, it is crucial to identify physical and cognitive limitations pertaining to daily living. Cognitive fatigue has shown to adversely impact neuromuscular function in younger adults, however its impact on neuromuscular fatigue, and associated brain function changes, in older adults is not well understood. The aim of the study was to examine the impact of cognitive fatigue on neuromuscular fatigue and associated prefrontal cortex (PFC) activation patterns in older women.

**Methods:**

Eleven older (75.82 (7.4) years) females attended two sessions and performed intermittent handgrip exercises at 30 % maximum voluntary contraction (MVC) until voluntary exhaustion after a 60-min control (watching documentary) and 60-min cognitive fatigue (performing Stroop Color Word and 1-Back tests) condition. Dependent measures included endurance time, strength loss, PFC activity (measured using fNIRS), force fluctuations, muscle activity, cardiovascular responses, and perceived discomfort.

**Results:**

Participants perceived greater cognitive fatigue after the 60-min cognitive fatigue condition when compared to the control condition. While neuromuscular fatigue outcomes (i.e., endurance time, strength loss, perceived discomfort), force fluctuations, and muscle activity were similar across both the control and cognitive fatigue conditions, greater decrements in PFC activity during neuromuscular fatigue development after the cognitive fatigue condition were observed when compared to the control condition.

**Conclusion:**

Despite similar neuromuscular outcomes, cognitive fatigue was associated with blunted PFC activation during the handgrip fatiguing exercise that may be indicative of neural adaptation with aging in an effort to maintain motor performance. Examining the relationship between cognitive fatigue and neuromuscular output by imaging other motor-related brain regions are needed to provide a better understanding of age-related compensatory adaptations to perform daily tasks that involve some levels of cognitive demand and physical exercise, especially when older adults experience them sequentially.

## Background

Fatigue is associated with impairments in both physical and cognitive functioning and interference in performing daily tasks [1–3]. It refers to normal, everyday experiences that are observed after sustained physical activity or mental exertion [1, 4, 5]. Specific to physical functioning, neuromuscular fatigue can be defined as a reduced ability to generate a desired muscle force [2, 6]. On the other hand, cognitive fatigue occurs due to prolonged periods of performing mentally demanding task that induces a state of subjective fatigue, feelings of “tiredness and lack of energy”, and performance decrements [2, 7]. Recent studies have elucidated the negative impact of cognitive fatigue on neuromuscular function, which includes changes in strength, muscle activity, and joint steadiness [7–12]. Concomitant to these biomechanical outcomes, cognitive fatigue has shown to limit activation of the prefrontal cortex (PFC) during muscle fatigue development in healthy young adults [12, 13]. Aging has shown to further exacerbate the impact of concurrent cognitive demand on biomechanical indicators of neuromuscular control in older adults during non-fatiguing tasks [14]. Because the normal aging process influences both physical and cognitive fatigue processes, it is likely that the nature and extent to which cognitive fatigue affects neuromuscular fatigue development in older adults differs that from younger adults; however there are no published biomechanical or neuroimaging data.

One of the vital functions related to functional independence and activities of daily living (ADL) in the geriatric population is the ability to grasp and hold objects [15]. This ability requires a complex series of inputs from various body systems including the central nervous system (CNS) as well as the musculoskeletal system [8, 16]. With advancing age, there are normal structural and functional changes that occur in the brain and within the musculoskeletal system, which may impact grasping functions. Older adults exhibit general decrements in grasping/holding due to age-related reduction in grip strength [16]. However, aging is associated with an increase in type I muscle fibers that has shown to increase fatigue resistance in older adults [17, 18]. Age-related changes at the CNS, particularly the brain, include reduced cerebral blood flow, loss of cortical excitability, reduction in cortical plasticity, and loss of grey matter [8, 19]. Subsequently, functional brain imaging studies have shown age-related increased compensatory activation at the PFC, ipsilateral cortical motor and sensorimotor areas to maintain motor performance [19, 20]. Conversely, reduced brain activity or inefficient cortical connectivity is observed in these brain regions, particularly the PFC, in older adults during fatiguing exercises (i.e. when motor task performance is not maintained) suggesting that PFC may be a limiting factor for impaired neuromuscular performance [21, 22]. Cognitive fatigue, like physical fatigue, has shown to adversely impact numerous cognitive functions including attention, working memory and executive control, and this is particularly evident in older adults [5, 23]. Working memory function (i.e., the ability to process and store information) is largely regulated by the PFC and is typically one of the first functions to deteriorate with age [24–27]. In older adults, working memory function is a crucial cognitive function that along with reasoning, language, and learning abilities, enables performance of ADLs [24]. At higher working memory loads, additional cognitive resources are required in order to compensate for the age-related declines in working memory function, specifically with reduction in activation of dorsomedial PFC [27].

Given that the PFC plays a major role in regulating both cognitive and neuromuscular abilities, and that the normal aging process impacts PFC functioning, it is likely that age-related changes in PFC activity may moderate neuromuscular function when individuals are both cognitively and physically fatigued. The purpose of this study was to examine the impact cognitive fatigue on neuromuscular fatigue and associated PFC activity in older adults. It was hypothesized that cognitive fatigue will influence neuromuscular fatigue development and this relationship will be associated with altered PFC patterns.

## Methods

### Participants

Eleven older females from the local community participated in this study. Participant demographics are presented in Table 1. All participants were screened for color-blindness, and self-reported right-handedness, sedentary lifestyles (less than 3 workouts of 30 min each in a week), and musculoskeletal injuries of the upper extremity currently or within the past year. Participants were also screened for cognitive impairments using the Mini-Cog™ test [28]. Written informed consent, using approved procedures by the Texas A&M University institutional review board was obtained from all participants.

**Table 1:** Participant demographic data

	Mean (SD)
Age (years)	75.82 (7.4)
Height (m)	1.59 (0.11)
Weight (kg)	78.65 (15.6)
BMI (kg/m^2^)	29.74 (4.9)
Handgrip strength (kg)	8.78 (2.5)
% Cognitively impaired (as screening using the Mini-Cog^TM^)	0 %
% Participants sedentary (defined as less than 3 workouts of 30 min each in a week)	100 %

### Experimental protocol

**Fig. 1:**
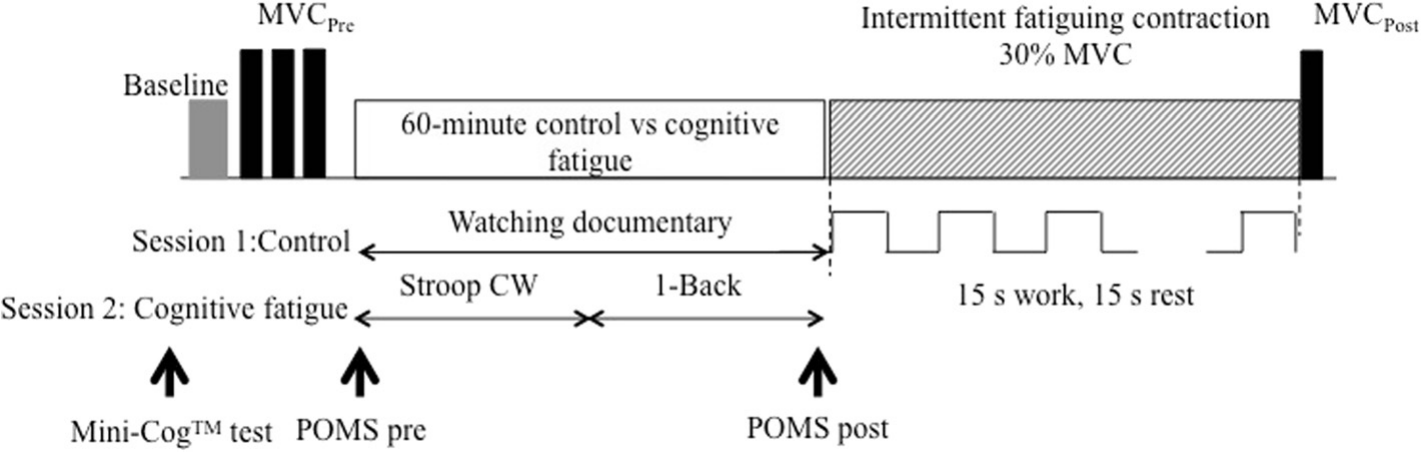
Experimental Protocol. The top panel illustrates the order in which the tasks were performed. The Mini-Cog^TM^ test was performed before the baseline to screen for cognitive impairment. Maximum voluntary contractions (MVC; solid bars) were performed at the beginning of data collection and immediately after the fatiguing contraction. Participants underwent a 60-min control condition (watching documentary) and cognitive fatigue condition (Stroop Color Word and 1-Back tests) that were counterbalanced on two separate days. POMS fatigue responses were obtained before and after the 60-min task to assess perceived cognitive fatigue levels. Following the 60-min task, participants performed intermittent submaximal handgrip fatiguing exercise at 30 % MVC (hashed rectangle) until volutnary exhaustion

A 2 task (Control vs Cognitive Fatigue) repeated measures model was employed to investigate the impact of cognitive fatigue on neuromuscular function and associated PFC activity in older females. Participants attended two experimental sessions that were counterbalanced and separated by at least 48 h to minimize order, learning, and fatigue effects. At the start of the first experimental session, participants were familiarized with the protocol and instructed to perform a series of warm-up handgrip exercises. This included intermittently gripping a stress ball for ~3 min. After adequate warm-up, participants were instrumented with various biosensors described later. Once instrumented, a 2-min functional baseline was collected where participants were instructed to focus on red mark placed within their seated eye-level to obtain baseline fNIRS data. After the functional baseline, a series of at least three maximum voluntary contractions (MVCs) were collected to obtain maximum handgrip strength using a digital handgrip dynamometer (BIOPAC Systems, Inc., Santa Barbara, CA, USA) with sufficient rest periods of ~2 min between each collection, in a seated posture. Participants were seated upright with their upper arm at their side with the elbow at 90 degrees and lower arm strapped to the chair arm support. The dynamometer was held in the dominant hand and the participant maintained the standardized grip testing posture. The experimental task required participants to intermittently perform handgrip exercises at 30 % MVC for 15 seconds (i.e., work cycle) with 15 seconds of rest between each gripping action until *voluntary* exhaustion, in the absence (control) and after one-hour cognitive fatigue exposure (Fig. 1). Data acquisition from all measurement systems, detailed later, was synchronized manually to a single start point. During both experimental sessions, the order of which was counter-balanced, participants were instructed to perform the handgrip exercises for as long as they could and precisely track their generated force against the target as closely as possible based on real-time visual feedback on a computer screen at eye height.

In the control condition, participants underwent a 60-min quiet session, where they watched a documentary and/or read a magazine. The cognitive fatigue condition was composed of two tests of 30 min each: 1) Stroop Color Word (CW) test and 2) 1-Back test. The Stroop CW and 1-Back tasks were chosen to target basic working memory functions and have shown to induce cognitive fatigue when performed for a longer duration [27, 29–31]. The Stroop CW test was composed of 3 blocks: 1) color 2) word, and 3) color-word. During the 1-Back test, participants responded only when the letter that was currently on the display was the same as the preceding letter. Throughout the 60-min cognitive fatigue task, subjective reports were verbally obtained to verify that participants were cognitively fatigued. For the purpose of this study, cognitive fatigue was operationally defined as increase in self-reported Profile of Moods States (POMS) fatigue subscale score [32]. The POMS fatigue subscale has been previously used to quantify perceived fatigue [7, 27]. The POMS fatigue subscale is composed of a series of 7 adjectives and is rated on a 0–4 scale (0 - *not at all* to 4 - *extremely*) for a total possible score of 28. POMS scores were obtained twice: 1) pre 60-min (Pre_CF_) control or cognitive fatigue and 2) post 60-min (Post_CF_) control or cognitive fatigue. In addition to the POMS fatigue subscale, percent correct responses from the Stroop CW and 1-Back tests were obtained every 10 min. In general, decline in task performance over time is also indicative of cognitive fatigue [33].

### Neuromuscular fatigue outcomes

Endurance time (ET) was determined as the time until voluntary exhaustion and was calculated when participants were unable to maintain the task intensity at 30 % ± 5 % MVC for at least 3 seconds. Strength loss, regarded as the “gold standard” of muscle fatigue, was used to assess neuromuscular fatigue [34], whereby a decrease in strength is indicative of neuromuscular fatigue. The modified Borg CR-10 scale [35] was used to collect Ratings of Perceived Discomfort (RPDs) on a 0–10 scale (0 - *not at all* to 10 - *extreme discomfort*). Participants were asked to report RPD every two minutes during the fatiguing exercise, and the rate of change for RPD was obtained for each condition. An increase in RPD rate is indicative of increased discomfort due to fatigue [36].

### PFC activity

PFC activity was measured using functional near infrared spectroscopy (fNIRS). fNIRS sensors (NIRO 200 NX, Hamamatsu Photonics, Japan) containing one emitter and one detector, 3 cm apart, were placed bilaterally on the participant’s forehead, between Fpz and Fp3 for the left hemisphere and between Fpz and Fp4 for the right hemisphere to obtain Fp1 and Fp2 regions according to the International EEG 10–20 system. The sensors were covered with a black headband to eliminate external lights. fNIRS data were collected at 5 Hz at near-infrared light at wavelengths of 735, 810, and 850 nm. Based on the manufacturer’s guidelines, the fNIRS system was zero-set at the beginning of the experiment and all future data were presented relative to the zero-set values. Changes in oxygenated hemoglobin (HbO_2_) and total hemoglobin (HbT) were recorded continuously from initial values. To obtain baseline cerebral oxygenation levels, participants performed a functional baseline task, where they were instructed to direct their attention to a red cross on the wall in front of them for 15 s. Maximum HbO_2_ and HbT values for each work cycle were determined using the middle 10-second window, as these values are indicative of the time with the strongest neuromuscular coupling [37]. In order to examine fNIRS data associated with fatigue progression across all participants, and because endurance times may vary between participants, the total number of work cycles was divided in five time stages and maximum HbO_2_ and HbT were averaged across each time stage, namely 1^st^ (initial), 2^nd^, 3^rd^, 4^th^, and 5^th^ (exhaustion). The baseline HbO_2_ and HbT values were subtracted from their respective task-related value, and normalized values (∆HbO_2_ and ∆HbT) across the five stages were analyzed statistically. Given evidence that HbO_2_ is a more robust and reliable measure of brain activation than HbT [38, 39], it was used as the main measure of functional brain activity.

### Muscle activity

Surface electromyography (EMG) was used to examine muscle fatigue development. Surface EMG electrodes (BIOPAC Systems, Inc., Santa Barbara, CA, USA) were placed on the lower arm muscles (flexor carpi radialis (FCR) and the extensor carpi radialis (ECR)). These muscles were chosen as they have shown to be activated during handgrip actions [40, 41]. The muscle sites were shaved and cleansed to reduce skin resistance prior to placement surface EMG electrodes. Surface EMG (Ag/AgCl) electrodes were placed along the belly of the FCR and ECR in the direction of the muscle fiber with a fix inter-electrode distance measured between the centers of the electrodes of 30 mm. After 20 min of stabilization, a quiet EMG trial was collected to remove signal bias. All raw EMG signals were band-passed filtered (20–450 Hz) in hardware, sampled at 1000 Hz, full-wave rectified, then corrected for resting levels, low-pass filtered (dual pass, 4th order Butterworth filter with effective cutoff frequency of 3 Hz), and normalized to maximal values (obtained from maximal handgrip exertions). Root mean squared (EMG RMS) values for each muscle were quantified by averaging across the 10-seconds windows, across the five time stages of each fatiguing exercise, using the same approach employed to analyze fNIRS data. To obtain the power spectrum, median power frequency (EMG mPF) was calculated within 10 s windows across the five time stages of the raw EMG signal in each condition using a Hamming window and fast-Fourier transform. Increases in EMG RMS and decreases in EMG mPF over time have shown to be strong indicators of muscular fatigue [42, 43].

### Force fluctuations

Force fluctuations were measured (i.e., standard deviation/mean force * 100) during the fatiguing handgrip exercises using force output from the handgrip dynamometer. Data was collected at 1000 Hz and recorded continuously during the duration of the task. The middle 10-second window of the work cycle was averaged across each of the five time stages. An increase in the force fluctuations has shown to be associated with fatigue progression [44].

### Cardiovascular responses

Cardiovascular measures included heart rate (HR) and heart rate variability (HRV). A 3-electrode electrocardiogram based on the III-lead configuration was used to obtain HR and HRV low/high frequency ratio (LF/HF). Data was collected at 1000 Hz and continuously throughout each condition. HR was recorded as beat-to-beat (R-R) intervals and average across the middle 10-second window of the work cycle was averaged across each of the five time stages. While increases in HR are observed during increased physical and cognitive workload [45, 46], increases in HRV LF/HF is indicative of sympathetic activation in response to cognitive stress [47].

### Statistical analyses

In order to verify that the Stroop CW and 1-Back tests successfully induced cognitive fatigue, a two-way repeated measures analysis of variance (RANOVA) was performed to determine the effects of task (Control vs. Cognitive Fatigue) and time (Pre_CF_ vs. Post_CF_) on POMS scores. A one-way RANOVA was performed to determine the effects of task (Control vs. Cognitive Fatigue) on endurance time and RPD scores. A 2-way RANOVA was performed on handgrip strength to determine fatigue development in both control and cognitive fatigue conditions. A 3-way RANOVA was performed to determine the effects of task (Control vs. Cognitive Fatigue), hemisphere (Right vs. Left), and time (5 time stages) on ∆HbO_2_ and ∆HbT. Additionally, separate RANOVAs were conducted to determine the effects of task (Control vs. Cognitive Fatigue) and time (5 time stages) on EMG RMS, EMG mF, force fluctuations, HR, and HRV. Statistical significance was determined when α < 0.05. Significant interaction effects were examined using pairwise comparisons with Bonferroni corrections as required. All statistical analyses were conducted using SPSS 22 (IBM SPSS Statistics, IBM Corp., Armonk, NY, USA). Summary data is presented as mean (SD).

## Results

**Fig. 2:**
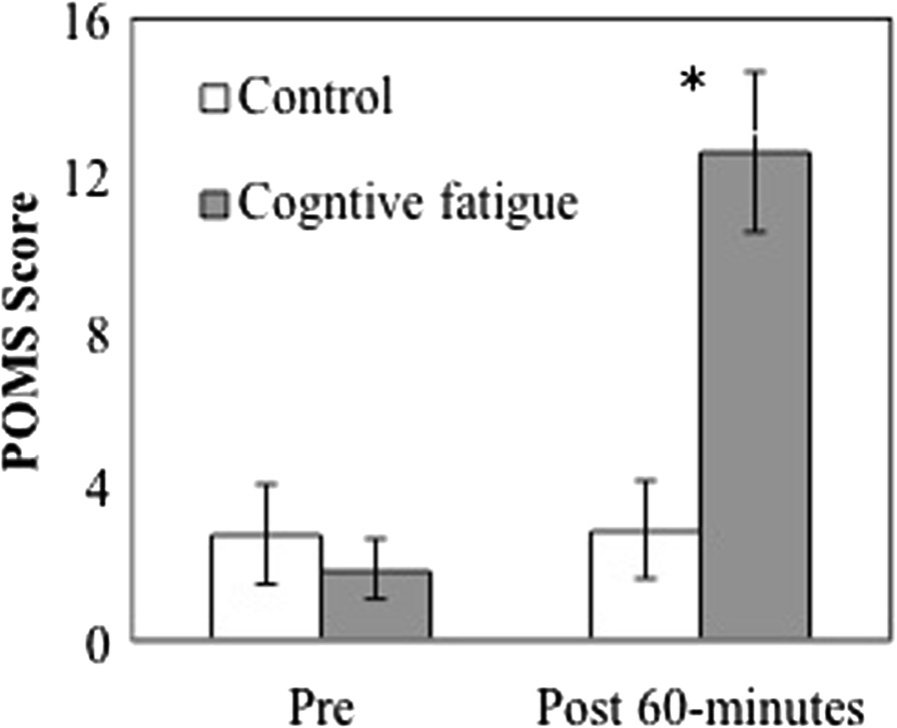
POMS scores pre and post 60 min cognitive fatigue conditions. * denotes significant difference (*p* < 0.05) between the control and cognitive fatigue condition. Error bars represent SE

POMS scores increased significantly over time (F_(1,10)_ = 33.21, *p* < 0.001, ƞ_p_^2^ = 0.769) and after the 60-min cognitive fatigue condition when compared to the control condition (F_(1,10)_ = 12.66, *p* = 0.005, ƞ_p_^2^ = 0.559; Fig. 2). Moreover, the interaction between task x time (F_(1,10)_ = 18.67, *p* = 0.002, ƞ_p_^2^ = 0.651) indicated that the increase in POMS score over time was only observed in the cognitive fatigue condition. The pre POMS score was similar across both the control and cognitive fatigue condition. Additionally, task performance trends in the one-hour cognitive fatigue task indicated general declines; percent correct responses decreased from 98.21 (0.12) % to 92.82 (0.15) % in the Stroop CW and 95.17 (0.04) % to 93.24 (0.05) % in the 1-Back test over time.

### Neuromuscular fatigue outcomes

Participants demonstrated 18.7 % decrease in endurance times when compared to the control condition (Control: 27.67 (21.39) min), however this difference was not significant (*p* > 0.141). Additionally, the rate of change for RPD was similar (*p* = 0.531) between the control (0.91 (1.14) /min) and cognitive fatigue condition (0.97 (1.43) /min). Baseline handgrip strength was similar across both the control and cognitive fatigue conditions (*p* = 0.439). A main effect of fatigue on strength was found (F_(1,10)_ = 24.39, *p* = 0.001, ƞ_p_^2^ = 0.709); the fatiguing exercises were associated with ~32 % decrements in handgrip strength. However, strength loss remained comparable across the control and cognitive fatigue conditions (*p* = 0.879).

### PFC activity

**Fig. 3:**
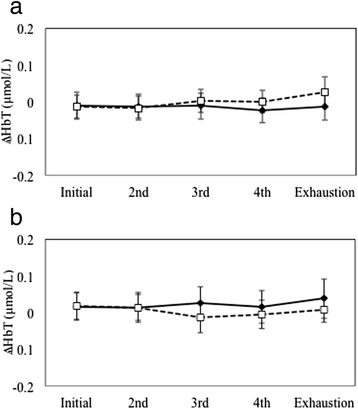
∆HbT levels in the cognitive fatigue condition (dashed lines) when compared to the control condition (solid lines) over time in the left (**a**) and right (**b**) PFC. Error bars represent SE

**Fig. 4:**
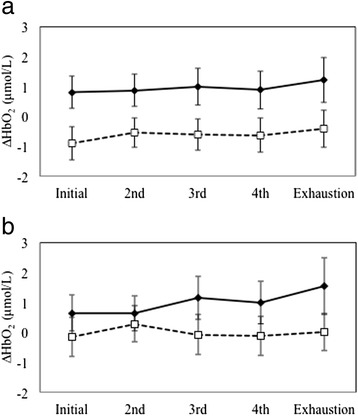
∆HbO_2_ levels in the cognitive fatigue condition (dashed lines) when compared to the control condition (solid lines) over time in the left (**a**) and right (**b**) PFC. Error bars represent SE

∆HbT values were not significantly affected by task, hemisphere, and time or any two- or three-way interactions (all *p* > 0.118; Fig. 3a, b). Since ∆HbT remained stable over time and across tasks, further analyses of brain activity were performed using ∆HbO_2_. The main effects of time (*p* = 0.108) and hemisphere (*p* = 0.501) on ∆HbO_2_ were not significant. However, a main effect of task (F_(1,10)_ = 6.49, *p* = 0.029, ƞ_p_^2^ = 0.394) on ∆HbO_2_ was observed; the 60-min cognitive fatigue exposure was associated with decreased PFC activity during the handgrip exercises when compared to the control condition. No significant differences in ∆HbO_2_ for any two-way interactions between task, hemisphere, or time were observed (all *p* > 0.231). However, the three-way interaction significantly influenced ∆HbO_2_ (F_(4,40)_ = 3.384, *p* = 0.018, ƞ_p_^2^ = 0.253; Fig. 4a, b). Post hoc comparisons between ∆HbO_2_ levels in the cognitive fatigue and control condition at each time stage and hemisphere did not show significant differences at *p*_*bc*_ < 0.005 (bonferroni correction for multiple comparisons). Figure 4a, b illustrate bilateral ∆HbO_2_ patterns over time across both tasks; activation was found to be greater in the control when compared to the cognitive fatigue condition across all time stages in both hemispheres, except at the 1^st^ and 2^nd^ stage in the right PFC.

### Muscle activity

EMG RMS of the FCR muscle increased significantly over time (F_(4,40)_ = 3.81, *p* = 0.028, ƞ_p_^2^ = 0.312; Table 2). Post hoc comparisons revealed greater activation observed at the 5^th^ stage when compared to the 2^nd^ (*p* = 0.03) and 3^rd^ stage (*p* = 0.019). There were no main effects observed for task, or its interaction with time, on EMG RMS for both muscles (all *p* > 0.152). Similarly, EMG mPF for both FCR and ECR muscles were not influenced by time, task, or their interaction (all *p* > 0.212).

**Table 2:** Summary data (Mean (SD)) of muscle activity, force fluctuation, and cardiovascular responses over time across the control and cognitive fatigue conditions

		Control					Cognitive fatigue				
		Initial	2nd	3rd	4th	Exhaustion	Initial	2nd	3rd	4th	Exhaustion
EMG RMS of FCR (% MVC)*	Mean	23.99	23.35	24.73	27.21	30.12	19.09	19.79	18.72	21.39	21.25
	SD	9.74	13.24	12.26	12.04	11.22	9.34	10.93	12.47	11.20	12.28
EMG RMS of ECR (% MVC)	Mean	41.33	42.82	40.82	44.34	44.50	33.56	32.11	30.51	33.51	34.44
	SD	15.45	18.23	16.63	18.62	18.40	11.60	11.58	12.95	13.77	13.36
EMG mPF of FCR	Mean	130.33	130.51	130.31	128.46	126.24	125.80	133.54	130.85	130.58	129.87
	SD	16.72	19.55	19.46	13.18	24.69	34.72	26.85	26.36	26.14	26.50
EMG mPF of ECR	Mean	178.86	177.62	178.03	177.52	178.18	177.84	176.18	178.51	179.26	179.10
	SD	11.94	11.36	9.00	9.79	10.95	11.27	11.89	8.94	10.53	11.70
Force fluctuations (%)*	Mean	4.63	3.59	3.41	4.37	4.58	4.20	3.95	3.01	3.35	5.69
	SD	2.30	1.39	1.52	2.38	2.66	2.37	2.82	0.96	1.23	4.21
HR (bpm)	Mean	84.49	84.02	82.72	91.45	83.26	83.56	82.72	85.98	84.45	84.19
	SD	25.21	20.00	16.42	19.31	14.80	22.93	18.75	28.02	27.35	21.95
HRV LF/HF	Mean	0.42	0.42	0.42	0.42	0.42	0.42	0.42	0.42	0.42	0.42
	SD	0.003	0.002	0.002	0.006	0.002	0.002	0.005	0.006	0.003	0.003

*denotes a main effect (*p* < 0.05) of time

### Force fluctuations

A main effect of time was observed (F_(4,40)_ = 2.88, *p* = 0.041, ƞ_p_^2^ = 0.292; Table 2), with greater fluctuations observed at the 5^th^ stage when compared to the 2^nd^, 3^rd^, and 4^th^ stage. No significant effects of task (*p* = 0.386) or task x time interaction (*p* = 0.973) on force fluctuation were observed.

### Cardiovascular responses

While HR increased over time (F_(4,40)_ = 3.083, *p* = 0.032, ƞ_p_^2^ = 0.306; Table 2), it was similar across both the control and cognitive fatigue conditions (*p* = 0.295). Additionally, no two-way interaction between task and time was observed (*p* = 0.261). Time, task, or their interaction, did not affect HRV LF/HF values (all *p* > 0.392).

## Discussion

The present study investigated the impact of cognitive fatigue on neuromuscular fatigue development and associated PFC activation patterns during submaximal fatiguing handgrip exercises in older females. The results indicated that while cognitive fatigue did not affect traditional indicators of neuromuscular fatigue, i.e., endurance time and strength loss, it was associated with greater decrements in ∆HbO_2_ levels in the PFC during neuromuscular fatigue development after the 60-min cognitive fatigue condition when compared to the control condition.

Neuromuscular capacity, measured as a function of endurance time, has shown to be negatively affected by cognitive stress *prior to* [7] and *during* physical fatigue exercises [9] in younger adults. In the current study, prior cognitively fatigued state did not affect endurance time in older females. Methodological differences may explain the varied outcomes in the aforementioned studies when compared to the present study. For example, Marcora et al. [7] investigated the effects whole body fatigue using a cycling fatigue protocol after completing a 90-min continuous performance task that required sustained attention, working memory, response inhibition, and error monitoring, while the current study focused on localized muscle fatigue of the lower arm after a 60-min exposure to two working memory tasks. Moreover, previous studies [7, 45] that have investigated the impact of cognitive fatigue on muscle capacity have focused on postural (shoulder) and lower extremity (quadriceps) muscles rather than smaller muscle groups employed during handgrip exercises. Thus it is likely that the impact of cognitive fatigue on neuromuscular capacity is task- and muscle-dependent, as previously reported by Mehta et al. [46, 48]. A recent study demonstrated that high levels of concurrent cognitive demand increases force fluctuations of the lower arm in older adults [49]. It was suggested that the increase in fluctuations in the older adults may have been amplified due to age-related changes in the motorneuron pool via excitation or decreased inhibition when increased cognitive demand is present. Interestingly, the same research group demonstarted similar time to task failure during submaximal ankle dorsiflexion fatiguing exercises in the absence and presence of concurrent cognitive demands in older adults [50], which is similar to what was observed here.

Older adults exhibited decrements in ∆HbO_2_ levels in the PFC as a result of cognitive fatigue when compared to the control condition even though neuromuscular fatigue outcomes, i.e., endurance time and strength loss, were similar. In a previous (and similar) investigation in young adults [13], we found that concurrent mental fatigue during neuromuscular fatiguing protocol (concurrent condition) was associated with greater PFC activation initially when compared to the same fatiguing exercise in the absence of mental fatigue (control). This trend was explained by an initial need for additional resources placed by the cognitive demand in order to maintain both cognitive and motor task performance [13]. We also found a reversal trend at exhaustion; PFC activity during the concurrent condition was significantly lower than that observed in the control condition. This decrease was attributed, in parts, to compensatory activation in different brain regions given that endurance times remained comparable between the two conditions. The present study did not find the aforementioned PFC activation trends with older adults. First, PFC activity during the cognitive fatigue condition was lower than that during the control condition. Second, the reversal trend was not observed; cognitive fatigue-related decrements in ∆HbO_2_ were consistent over time. However, ∆HbO_2_ trends (Fig 4) suggest that the magnitude of the difference between control and cognitive fatigue-specific activation is both hemisphere- and time-dependent, whereas in our previous investigation in younger adults [13] we only observed time-dependent PFC activation patterns. It is likely that aging may differentially impact bilateral PFC regulation when individuals are both cognitively and physically fatigued. In support of this, previous research has demonstrated that age-related reductions in PFC activation is observed during physically fatiguing exercises [21, 22] as well as during tasks involving the working memory [51]. Interestingly, neuromuscular performance remained unchanged between the control and cognitive fatigue conditions, indicating potential neural adaptations to compensate for the observed reduction in PFC activity. The *scaffolding theory of cognitive aging* [52–55] suggests that with aging there is a compensatory shift in neural recruitment to accommodate cognitive challenge [54, 55]. While the present study focused solely on the activation of the PFC, other brain regions are involved in fatigue development, particularly in older adults [8, 19, 56]. Previous research has demonstrated a shift in activation centers in the brain to maintain neuromuscular performance during a fatiguing protocol [57]. It is likely that the presence of cognitive fatigue accelerated cortical redistribution in older adults to maintain neuromuscular performance. Because only the PFC was monitored in the present study, further investigation is warranted to provide support for the proposed hypothesis on shifting of brain activation centers when older adults are both physically and cognitively fatigued. The premotor and motor areas may be of interest due to their interconnection with the PFC during motor tasks under stress [58]. Particularly with aging, the PFC decreases in size, which may alter connection to the premotor cortex and result in a decline in motor capabilities [59].

In general, heart rate increased linearly during neuromuscular fatigue development, implying that all participants reached similar physiological fatigued states [47]. This physiological increase over time was accompanied with an increased perception of discomfort. While heart rate and ratings of perceived exertions are highly correlated [60], these outcomes were not found sensitive to the different cognitive fatigue conditions, which are a departure from findings reported by Marcora et al. (2009), who demonstrated that a prior cognitive fatigued state is associated with greater perception of effort and discomfort and decreased muscle endurance. It is likely that the exposure to the cognitively fatiguing task may have played a role in the discrepancies observed between the two studies. Heart rate variability, as measured by the LF/HF ratio (a measure of sympathovagal balance [61, 62]), was also found to be similar across both cognitive fatigue conditions in the present study. An increase in LF/HF has shown to indicative of increased stress [61, 63, 64]. Thus similar LF/HF ratios across both cognitive fatigue and control 60-min conditions indicate that the participants experienced similar levels of stress, if any, across both conditions.

There are some limitations in the present study that warrant discussion. First, the study examined the impact of cognitive fatigue on neuromuscular function and associated PFC activity in older females. This was done to avoid sex-specific differences in both cognitive and neuromuscular capacity. Pereira et al. [49] reported that older females are more susceptible to the effects of neuromuscular fatigue; especially at high and low levels of cognitive demand during sustained upper arm exertions, when compared with males of the same age. Additionally, they reported that younger women exhibited greater fatigability, at higher levels of cognitive demand, compared to their male counterparts. These sex differences may have important implications for work-related tasks that require either high or low levels of cognitive workload, particularly with the aging workforce. Future research is warranted to extent the current investigation to include a larger sample of both males and females in both the younger and older population to examine age-specific differences. Second, the present study monitored the PFC regions due to equipment constraints. Existing neuroimaging investigations of fatigue development suggest a shift in activation centers in the brain to compensate for fatigue-related loss in neural efficiency, particularly with the normal aging process [8, 19, 57]. Future research is needed to examine activation of motor function-related brain regions to understand age-related changes in functional brain activation patterns when individuals are physically and cognitively fatigued. Third, differentiating the effects of cognitive fatigue and stress and/or anxiety that may originate due to the cognitive fatigue protocol through the use of cortisol markers may provide a better understanding of how neuromuscular fatigue and associated PFC functioning is impacted by these non-biomechanical risk factors in older adults. Finally, a majority of the participants were overweight and obese (body mass index (BMI): 29.74 (4.9) kg/m^2^). Because increased adiposity has been previously associated with impaired neuromuscular functioning and altered brain function [36, 65, 66], it is likely that the BMI status of the study pool may have influenced the study outcomes. For example, in younger obese adults, altered handgrip force control has been associated with stunted PFC activity [65], particularly under stress [66]. However, there is no published evidence of the same in the geriatric population. Results from the present study need to be supplemented with data from non-obese and obese older males and females to examine how obesity influences the findings reported here.

## Conclusion

Concurrent mental fatigue and/or stress have previously shown to adversely impacts neuromuscular performance and muscle endurance in young adults. However, in the current study traditional biomechanical measures indicated that prior cognitive fatigue did not alter muscle endurance in older females. Using fNIRS, blunted PFC activation patterns were observed in response to cognitive fatigue, despite similar neuromuscular outcomes, which may be indicative of neural adaptation with aging in order to regulate the effects of cognitive fatigue on neuromuscular fatigue. Brain imaging investigations of other motor-related brain regions are needed to provide a better understanding of age-related compensatory adaptations to perform daily tasks that involve some levels of cognitive demand and physical exercise, especially when older adults experience them sequentially. The current findings suggest that promoting brain health in healthy aging interventions may help to improve the quality of life and physical capabilities in older adults.
